# Ultrasound assessment of intimal hyperplasia after plain balloon angioplasty and drug-coated balloon angioplasty of AV access stenosis

**DOI:** 10.1186/s42155-025-00621-3

**Published:** 2025-12-13

**Authors:** Kate Steiner, Clare Kirwan, Siva Ramanarayanan

**Affiliations:** Department of Radiology, The Lister Hospital, East and North Hertfordshire NHST, Corey’s Mill Lane, Stevenage, Hertfordshire SG4 4AB UK

**Keywords:** Arteriovenous fistula, AVF stenosis, Intimal hyperplasia, Drug-coated balloon angioplasty, Ultrasound assessment of intimal hyperplasia

## Abstract

**Purpose:**

To determine whether there is a significant decrease in intimal hyperplasia post percutaneous transluminal angioplasty (PTA) of AV access stenosis. Comparing drug-coated balloon (DCB) angioplasty with plain uncoated balloon (PUB) angioplasty by examining B-mode ultrasound measurements of percentage intimal medial thickening (%IMT) in stenotic lesions pre and post PTA.

**Methods:**

One hundred ninety-one consecutive PTA procedures for AV access dysfunction were screened retrospectively for inclusion. Those procedures where there was an ultrasound prior to and following PTA with measurements of IMT were included.

**Results:**

Ninety-nine stenotic lesions were included in a total of 87 patients. A total of 26/99, 26%, were treated by DCB angioplasty, and a total of 73/99, 74%, were treated by PUB angioplasty.

The difference between the pre-PTA and post-PTA %IMT was calculated and defined as the delta-%IMT for each group. There was a greater reduction in %IMT in the DCB group (mean delta-%IMT =  − 22.35%) when compared with the PUB group (mean delta-%IMT =  − 5.94%), *p* = 0.0005.

Delta-%IMT for those lesions where there was a baseline pre-PTA %IMT of greater than 25% was examined. The mean delta-%IMT reduced in the PUB group from − 5.94% to − 2.20% and remained similar in the DCB group at − 20.05%, *p* = 0.0003.

A Kaplan–Meir survival analysis examining primary patency over 24 months did not demonstrate any significant difference between the 2 groups.

**Conclusion:**

The statistically significant decrease in %IMT post PTA using a DCB compared with PUB angioplasty appears to demonstrate an anti-proliferative drug effect on lesion intimal hyperplasia. However, this did not translate into a sustained difference in target lesion primary patency.

**Graphical Abstract:**

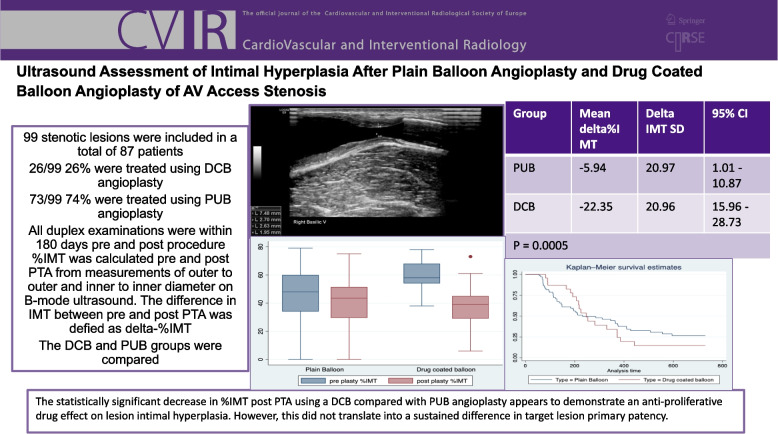

## Introduction

Failure of maturation, dialysis access dysfunction and thrombosis are most often caused by a haemodynamically significant stenosis in the access circuit [1]. Dialysis access thrombosis and dysfunction are one of the leading causes of morbidity and mortality among end stage kidney disease patients [2, 3]. Percutaneous trans-luminal balloon angioplasty (PTA) is a well-recognised, minimally invasive, effective and safe method used to treat arteriovenous (AV) access stenosis. However, primary patency rates following plain uncoated balloon (PUB) angioplasty are low at 6–12 months and continue to reduce over time, often resulting in multiple repeat interventions to maintain functional patency [4]. The evidence base for superior efficacy of DCB angioplasty over PUB angioplasty in AV access contains conflicting trial results and can be described as mixed [5–8]. For example, the IN.PACT AV Access Randomized Trial of Drug-Coated Balloons for Dysfunctional Arteriovenous Fistulae demonstrated higher primary patency rates for DCB interventions compared with PUB angioplasty [6, 8]. However, the paclitaxel-assisted balloon angioplasty of venous stenosis in haemodialysis access (PAVE) clinical study did not demonstrate significantly higher primary patency rates where DCBs were used compared with PUBs [7]. There are many differences between the trials including, patient populations, fistula maturity, trial design and DCB type which could contribute to the differences in trial outcomes. One other potential trial variable is AV access lesion morphology or type. Intimal hyperplasia is a well-recognised cause of AV access stenosis and can be identified on B-mode ultrasound imaging [9]. When examining AV access stenoses using ultrasound percentage intimal medial thickening (%IMT) can be measured and this appears to correlate with intimal hyperplasia on histology [9]. % IMT varies between lesions and a small percentage of lesions both pre and post PTA demonstrate no measurable IMT [10]. PTA results in endothelial and smooth muscle cell injury which causes migration of smooth muscle cells and myofibroblasts to the intima where they proliferate resulting in intimal hyperplasia. The antiproliferative agents used to coat DCBs could potentially target this process and therefore should result in a greater reduction in %IMT on ultrasound after DCB angioplasty when compared with PUB angioplasty. We have examined the reduction in %IMT measured on ultrasound examinations pre and post AV access intervention using DCBs and PUBs to assess whether there is any measurable significant antiproliferative effect of paclitaxel on %IMT post DCB angioplasty.

## Materials and methods

It is standard practice at our institution to perform a pre-procedural duplex ultrasound examination, which is requested to investigate the cause of dialysis access dysfunction. After PTA is performed, the patients are recalled for a post-procedural duplex ultrasound examination. Over 18 months, 191 consecutive percutaneous angioplasty (PTA) procedures for AV access dysfunction were screened retrospectively for inclusion. Those procedures where there was an ultrasound within 180 days prior to and within 180 days following PTA with measurements of IMT were included. Ultrasound examinations were performed in a dedicated vascular laboratory by a consultant interventional radiologist and two vascular trained sonographers. Patients were scanned in a sitting position using a GE (General Electric Healthcare, Little Chalfont, Buckinghamshire, UK) Logic E9 and a 15 or 9 MHz probe. Measurements of total venous diameter and luminal diameter were used to calculate %IMT using the formula:

$$Outer\;to\;outer\;wall\;vessel\;wall\;diameter\;-\;Luminal\;diameter\; \div\;Outer\;to\;outer\;wall\;vessel\;diameter\;X\;100\;=\%\;\text{IMT}$$

**Fig. 1:**
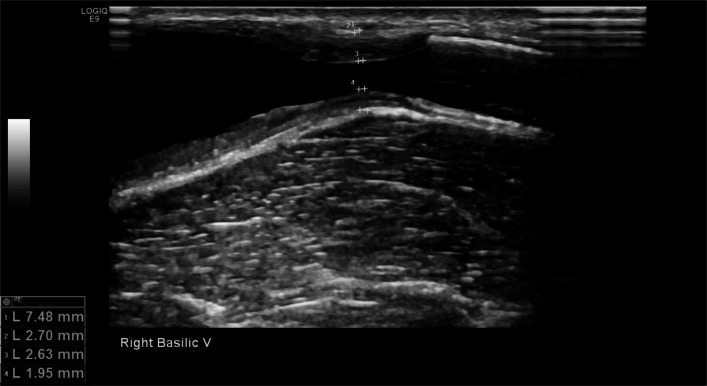
B-Mode ultrasound image demonstrating a basilic venous stenosis proximal to a stent graft. Measurements of outer-outer diameter, luminal diameter, near wall, and far wall IMT have been taken as shown

Measurements of near wall and far wall intimal medial thickness were also measured, this is standard practice at our institution as shown in Fig. 1. All duplex examinations are recorded on the radiology picture archiving and communication system (PACS).

The difference between the pre and post-PTA %IMT was calculated and compared between the DCB and PUB angioplasty groups. Patient demographics were recorded.

The drug-coated balloons used within the study were the Lutonix™ DCB catheter (Becton Dickinson) and Ranger™ balloon (Boston Scientific). For all PTA procedures, pre-dilation was performed using either a high pressure balloon, ultra-high pressure balloon or cutting balloon prior to DCB angioplasty. Procedures were performed by three consultant interventional radiologists. Inflation of the DCB was for a minimum of 120 s.

### Statistical analysis

Continuous variables were expressed as mean, standard deviation and categorical data were expressed as a percentage. The paired *T* test was used to assess statistical significance of change in % IMT pre and post treatment. A *p* value of < 0.05 was set as cut-off for statistical significance. Kaplan–Meir survival curves were used to estimate event free survival following PTA. All analysis were performed using STATA software (StataCorp. 2024. *Stata Statistical Software: Release 17*. College Station, TX: StataCorp LLC.)

## Results

A total of 99 stenotic lesion in 87 patients met the inclusion criteria. The majority of lesions excluded were excluded because there was no follow up ultrasound examination within the 180 days time frame of the study, or the lesion was located within the central veins and therefore could not be examined using ultrasound.

The majority of procedures were PUB angioplasty at 74% (73/99) and 26% (26/99) were DCB angioplasty. The pre-PTA duplex mean time of assessment was 45 days (range 0–159 days) and post PTA the mean time of assessment was 65 days (range 17–180 days).

### Baseline characteristics

The baseline characteristics are summarised in Table 1. The mean age and proportion of diabetics were similar across the PUB and the DCB group. However, the DCB group had a higher number of previous PTA procedures to the lesions, higher baseline %IMT prior to PTA and higher proportion of male patients and inflow lesions.

**Table 1:** Baseline characteristics

Variable	Plain uncoated balloon (*n* = 73)	Drug coated balloon (*n* = 26)
Age in years (mean +/− SD)	66.4 +/− 13	68.2 +/− 14.7
Gender (%male)	57.6	72.7
Diabetes (%)	59.9	59.1
Mean number of previous PTA procedures	1.3 +/− 2.5	3.7 +/− 2.8
%IMT pre PTA	44.2 +/− 22.9	59.9 +/− 10.5
AVF configuration		
Forearm (%)	65	60
Upper arm (%)	35	40
Location of lesion		
Inflow segment (%)	46	65
Cephalic vein outflow (%)	4	4
Cephalic arch (%)	41	23
Other (%) (basilic vein outflow/graft vein anastomosis)	9	8

### Analysis of %IMT response to plain balloon angioplasty compared with drug-coated balloon angioplasty

The majority of lesions had some measurable %IMT (91%), a small proportion (9%) had no measurable IMT pre-PTA.

**Fig. 2:**
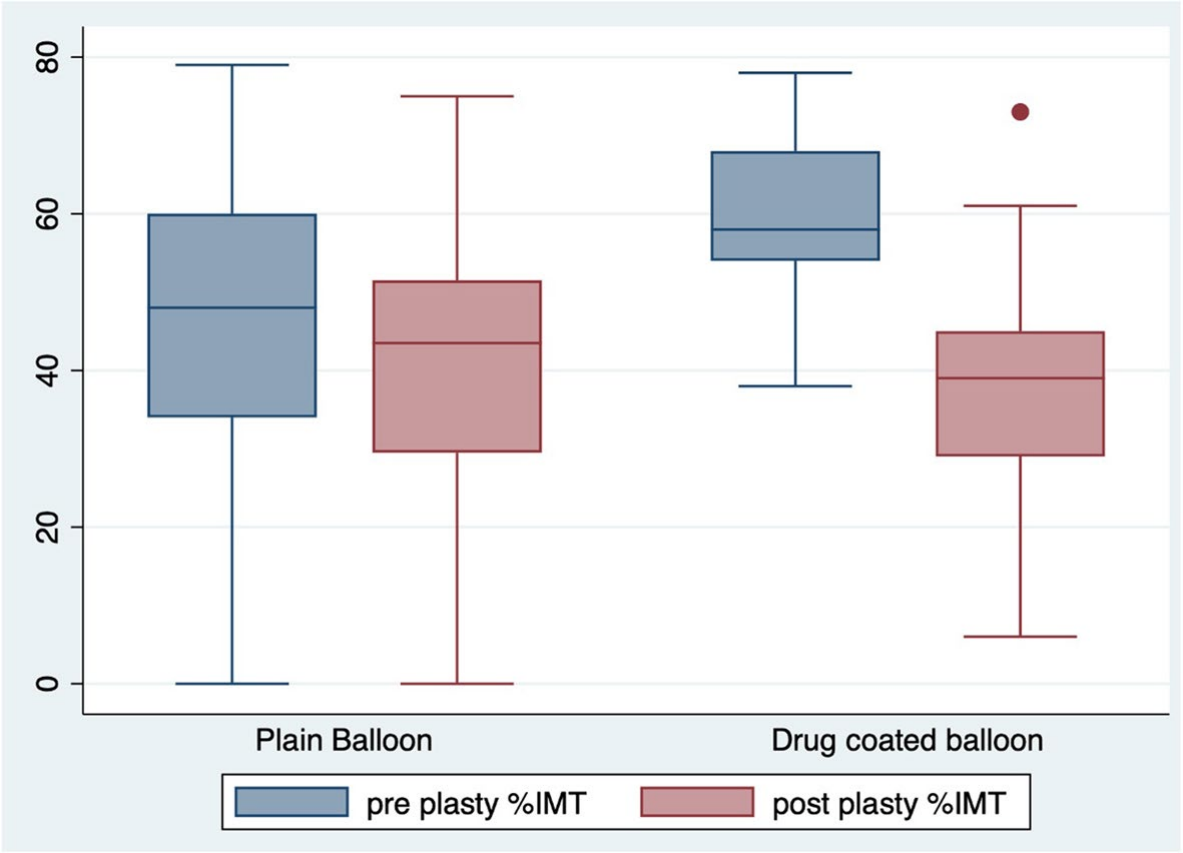
Box plot analysis. This demonstrates the median %IMT, upper and lower quartile %IMT comparing the PUB and DCB groups. There is a greater reduction in %IMT in the DCB group

The pre-PTA %IMT and post-PTA %IMT were compared between the two groups. A box plot analysis was performed which demonstrates a greater reduction in %IMT post PTA in the DCB group (Fig. 2).

The difference between the pre-PTA and post-PTA %IMT was calculated and defined as the delta-%IMT for each group. There was a greater reduction in the delta-%IMT in the DCB group − 22.35% (SD [20.97], 95% CI [1.01, 10.87]) when compared with the PUB group − 5.94%, (SD [20.96], 95% CI [15.96–28.73]). This was statistically significant *p* = 0.0005.

The mean delta-%IMT was compared between the DCB and PUB group for those lesions where there was a baseline pre-PTA %IMT of greater than 25%. This was done to avoid selection bias and makes both groups more comparable because there were a higher number of lesions with minimal or no measurable intimal hyperplasia in the PUB group. The mean delta-%IMT reduced in the PUB group from − 5.94% to − 2.20% (SD = [19.66], 95% CI [3.07, 7.46]) and remained similar in the DCB group at − 20.05% (SD = [15.47], 95% CI [13.19, 26.9]). The difference in mean delta-%IMT between the groups remained statistically significant *p* = 0.0003.

### Target lesion primary patency—Kaplan–Meir survival analysis

**Fig. 3:**
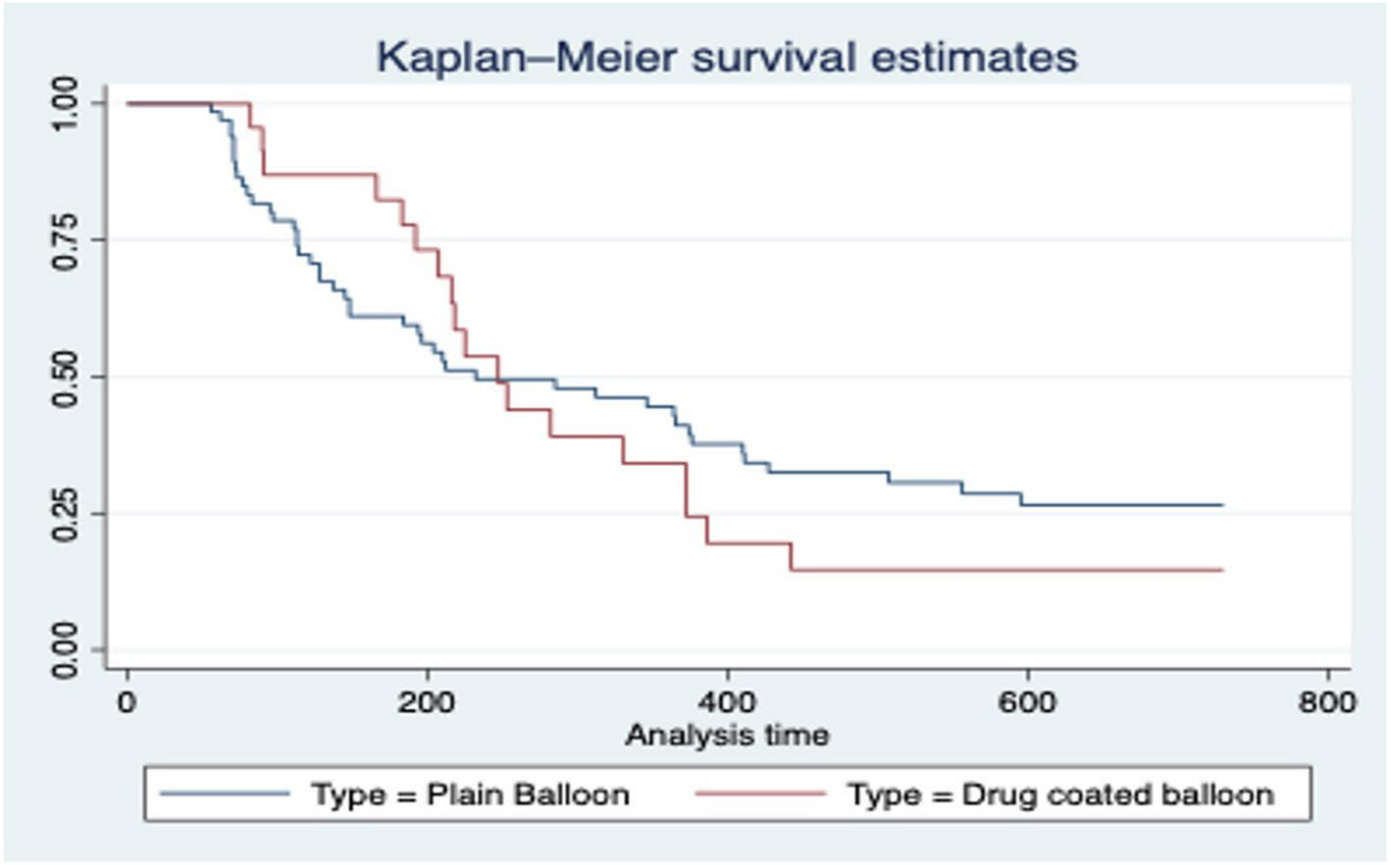
Kaplan–Meir survival curve comparing target lesion primary patency post venoplasty between PUB and DCB group (50% event free survival of 232 and 247 days respectively in PUB and DCB groups—statistically non-significant)

Target lesion primary patency was then compared between the PUB and DCB groups over a 24-month follow up period. The Kaplan–Meir survival analysis is shown in Fig. 3.

The average time interval at which 50% of lesions develop the event of interest is similar in both groups (232 days in the PUB group and 247 days in the DCB group) which was not statistically significant.

## Discussion

**Fig. 4:**
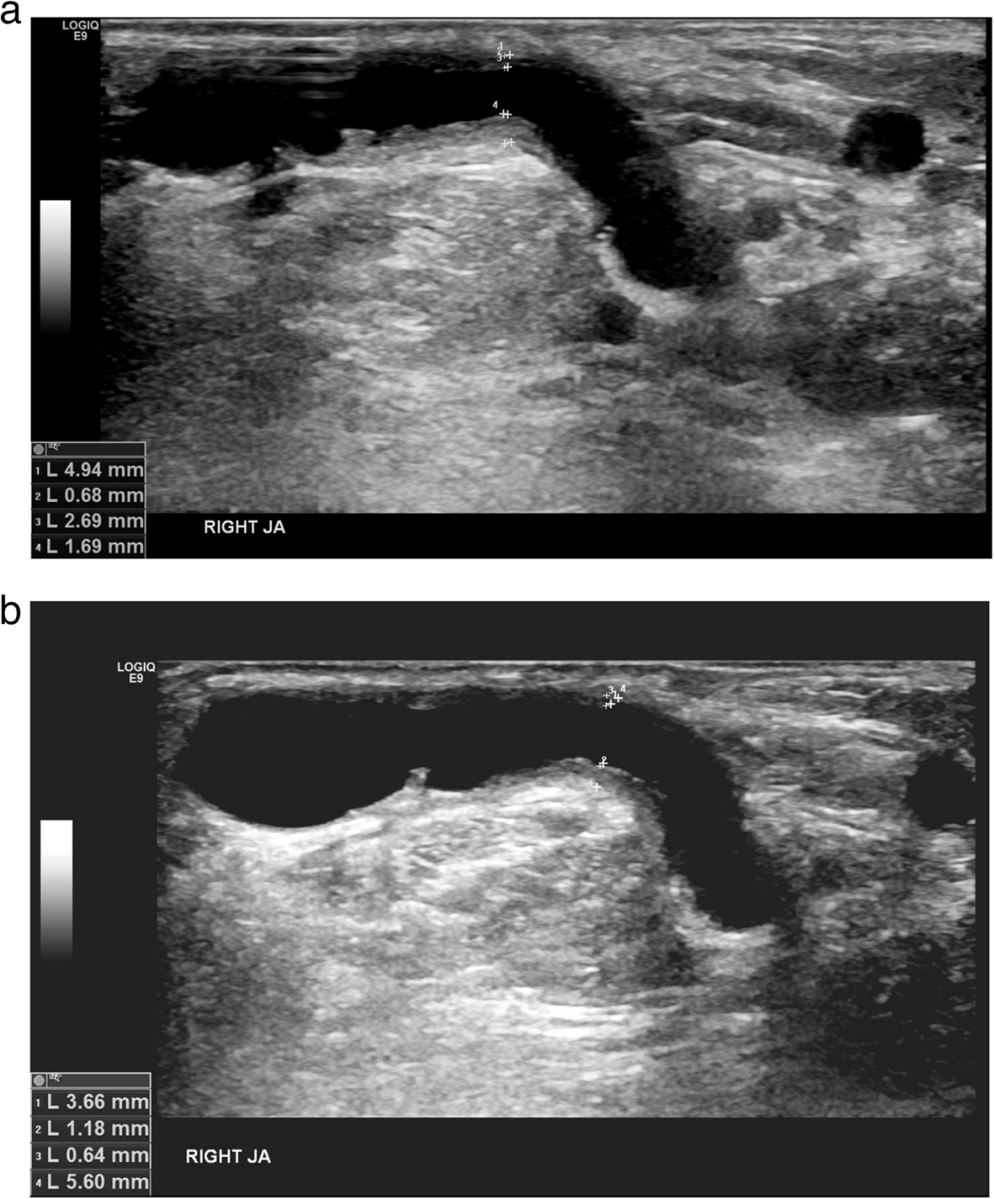
**a**, **b** B mode ultrasound images of a juxta-anastomotic stenosis pre (**a**) and 59 days post DCB angioplasty (**b**). %IMT has reduced from 50 to 33%

The study results demonstrate a statistically significant decrease in %IMT between the pre and post PTA ultrasound examinations for the DCB group when compared with the PUB balloon group. This demonstrates that a drug effect of paclitaxel on lesion intimal hyperplasia has been observed in this study, which would be predicted by its antiproliferative mechanism of action. An example of ultrasound appearances pre and post DCB angioplasty are shown in Fig. 4a, b. The reduction in mean delta-%IMT further decreased (from − 5.94% to − 2.02%) in the PUB group when lesions with no measurable IMT and a %IMT < 25% were excluded suggesting that PUB angioplasty had no significant effect on reducing %IMT post PTA.

Other groups have examined lesion morphology using ultrasound and how this may affect outcomes after endovascular treatments [11–14]. Wang et al. in 2024 examined lesion type based on ultrasound characteristics and demonstrated no significant different in patency rates following PTA when comparing predominantly intimal hyperplasia type lesions and non-predominant intimal hyperplasia type lesions [14]. They described five lesion types; intima-dominant, non-intima-dominant, valve obstruction, vascular calcification and mixed groups. Of these lesion types, only vascular calcification was a predictor of lower primary patency rates following PTA, and the other lesion types were not independent predictors of primary patency [14]. As yet, there is no consensus regarding classification of lesion type in AV access stenosis, criteria and description of stenosis type vary between studies which means that results are not directly comparable [10–14].

The significant decrease in %IMT observed in our study between the pre and post PTA ultrasound examinations seen in the DCB group did not translate to any statistically significant difference in lesion primary patency between the DCB and PUB group. The “late catch up phenomenum” where primary patency rates after DCB use declines in the long term, after initially demonstrating higher primary patency rates when compared with PUB angioplasty, has been well described in femoropoliteal arterial disease [15]. This is likely to be secondary to loss of antiproliferative drug effect over time resulting in cellular proliferation, neo-intimal hyperplasia and restenosis. The Kaplan–Meier analysis appears to demonstrate this in our study. Initially, higher primary patency rates are observed in the DCB group; after 223 days, the survival curves meet, and this is reversed, higher primary patency rates are observed in the PUB group.

The DCB group had had a larger number of previous interventions (3.7 +/− 2.8) compared with the PUB group (1.3 +/− 2.5), and there was only 1 de-novo lesion in the DCB group. This is an important factor to consider as it has been demonstrated that patency post PTA in AV access is affected by previous interventions, and primary patency after the first PTA is generally higher than after repeat procedures [13, 14, 16]. %IMT was also higher within the DCB group (59.9 +/− 10.5) compared with the PUB group (44.2 +/− 22.9). The demographics in the DCB group likely reflects generally adopted practice at our institution to use DCBs to treat recurrent lesions with ultrasound evidence of intimal hyperplasia; however, practice varied among operators, and recurrent lesions with no ultrasound evidence of intimal hyperplasia were also treated using DCBs. By selecting a patient population with a higher number of previous PTA procedures and %IMT, we may have selected a population which would be expected to have higher recurrence rates and lower primary patency rates.

In the future, ultrasound assessment of stenosis type together with consideration of the patients end stage kidney disease life plan as per the KDOQI 2019 guidelines may inform our choice of endovascular therapy [17]. At present, the conflicting trial results examining DCB efficacy and DCB cost have meant that their use is not as yet standard practice for all stenoses and to date there is no agreed treatment algorithm [18].

A cost analysis examining the 12-month data of the INPACT AV access study concluded that DCB use was potentially cost effective if follow up data supported assumptions of longer term clinical effectiveness [19]. A more tailored approach would potentially be more cost effective in the long term, if more costly endovascular therapies such as DCBs and stent grafts are used in a treatment algorithm which takes into account lesion %IMT or type, location, repeat interventions and other clinical variables.

## Study limitations

A retrospective analysis was performed which is subject to the limitations of a retrospective study. The two groups varied in lesion morphology, comparison was not between two uniform groups; the DCB group had a higher %IMT. Treatment in the PUB group was PUB angioplasty only, compared with the DCB group where PUB angioplasty was performed followed by a second inflation of the DCB, in the PUB group no second inflation was performed after treatment. This study is not powered to perform a sub-group analysis examining lesion location and DCB balloon type.

The post procedure ultrasound examinations were performed at a mean of 45 days and not a uniform time point within the study. There is no consensus, as yet, regarding timing of ultrasound follow up post PTA to examine drug effect. In the future, studies examining lesion morphology on ultrasound at regular time points over 24–36 months would provide more information on lesion response and restenosis.

## Conclusion

This study has demonstrated a statistically significant decrease in %IMT measured on ultrasound after DCB angioplasty when compared with PUB angioplasty. This appears to demonstrate a significant antiproliferative drug effect; however, it did not translate into improved primary patency rates after angioplasty where no significant difference was observed between the DCB and PUB groups.

Further assessment of lesion morphology prior to and after PTA in larger randomized control trials is warranted and is part of the trial protocol for selected sites in the Paclitaxel or sirolimus coated balloons for ArterioVEnous fistulas (PAVE-2 trial) which is currently recruiting in the UK [20].

## Data Availability

The excel spreadsheet containing the data collected is available to the publisher on request.
